# Effects of Intravenous Ketamine on Posttraumatic Stress Disorder (PTSD): A Systematic Review

**DOI:** 10.1111/acps.70053

**Published:** 2025-12-01

**Authors:** Liyang Yin, Andy Lu, Gia Han Le, Christine E. Dri, Sabrina Wong, Kayla M. Teopiz, Heidi Xu, Roger Ho, Taeho Greg Rhee, Heidi Ka Ying Lo, Maria‐Christina Sioufi, Yang Jing Zheng, Hezekiah C. T. Au, Hernan F. Guillen‐Burgos, Bing Cao, Roger S. McIntyre

**Affiliations:** ^1^ Brain and Cognition Discovery Foundation Toronto Ontario Canada; ^2^ Department of Psychology University of Western Ontario London Ontario Canada; ^3^ Institute of Medical Science, University of Toronto Toronto Ontario Canada; ^4^ Poul Hansen Family Centre for Depression University Health Network Toronto Ontario Canada; ^5^ Department of Pharmacology and Toxicology University of Toronto Toronto Ontario Canada; ^6^ Department of Psychological Medicine National University of Singapore Singapore Singapore; ^7^ Institute for Health Innovation and Technology (iHealthtech), National University of Singapore Singapore Singapore; ^8^ Division of Life Science (LIFS), Faculty of Science Hong Kong University of Science and Technology Hong Kong China; ^9^ Department of Psychiatry Yale School of Medicine New Haven Connecticut USA; ^10^ Department of Public Health Sciences University of Connecticut School of Medicine Farmington Connecticut USA; ^11^ Department of Psychiatry, School of Clinical Medicine, LKS Faculty of Medicine The University of Hong Kong Hong Kong China; ^12^ Department of Human Biology and Physiology University of Toronto Toronto Ontario Canada; ^13^ Joint Department of Medical Imaging University of Toronto Toronto Canada; ^14^ Division of Neurosurgery, Department of Surgery University Health Network and University of Toronto Toronto Ontario Canada; ^15^ Department of Psychiatry and Mental Health Pontificia Universidad Javeriana Bogota Colombia; ^16^ Center for Clinical and Translational Research Universidad Simon Bolivar Barranquilla Colombia; ^17^ Center for Clinical and Translational Research Bogota Colombia; ^18^ School of Psychology and Key Laboratory of Cognition and Personality (Ministry of Education), Southwest University Chongqing P. R. China; ^19^ Department of Psychiatry University of Toronto Toronto Ontario Canada

**Keywords:** Ketalar, ketamine, N‐methyl‐D‐aspartate (NMDA) receptor, post‐traumatic stress disorder, posttraumatic stress symptoms, trauma‐related stress

## Abstract

**Introduction:**

Posttraumatic stress disorder (PTSD) is a mental disorder resulting from exposure to traumatic events. Evidence suggests that ketamine may be efficacious in treating PTSD, however, ketamine's mechanisms in treating PTSD remain unclear. Herein, this review aims to evaluate the clinical outcomes of ketamine treatment in persons with PTSD and investigate the possible neurobiological mechanisms underlying ketamine's therapeutic effect in PTSD.

**Methods:**

A systematic search was conducted on PubMed and OVID (MEDLINE, Embase, PsychINFO) from inception until September 2025. Randomized controlled trials reporting on the effects of intravenous ketamine to treat PTSD were included.

**Results:**

Seven studies with a total of 323 participants were included in this review. Ketamine administration meaningfully improved PTSD symptoms in two trials as evidenced by significant improvement on the Clinician‐Administered PTSD Scale for DSM‐5 (CAPS‐5) and the Impact of Event Scale‐Revised (IES‐R) compared to control/placebo. Multi‐infusion administration schedules achieved greater clinical outcomes when compared to single‐dose administration schedules. Preliminary evidence suggests that repeated lower doses (0.2mg/kg) of ketamine were more efficacious in sustaining treatment effects than standard doses (0.5mg/kg). For persons receiving ketamine, an association was observed between top‐down inhibition of the amygdala originating in the ventromedial prefrontal cortex (vmPFC) and symptom improvement.

**Conclusion:**

Our results suggest that intravenous ketamine may be efficacious in the treatment of PTSD. Subsequent studies should attempt to evaluate the additive effect of combining ketamine with psychotherapeutic interventions as well as determining mechanistic pathways mediating symptom relief in persons with PTSD.

## Summary

- Summations
  ○ Intravenous ketamine administered in certain treatment regimens may be efficacious in the treatment of adults with PTSD.
  ○ Ketamine's mechanisms in treating PTSD remain unclear and require further investigation.
- Limitations
  ○ The methodologies of extant randomized controlled trials which evaluate ketamine in PTSD vary significantly.
  ○ The absence of available literature precluded analysis of ketamine's long‐term treatment effects for PTSD.

## 1 Introduction

Posttraumatic stress disorder (PTSD) is a mental disorder resulting from exposure to actual and/or threatened traumatic events and is associated with significant distress, suffering, as well as impairments in daily functioning and well‐being [1, 2]. PTSD is characterized by symptoms across four discrete subdomains of psychopathology, including intrusion, avoidance, negative alterations in cognition and mood, and changes in arousal and reactivity [3, 4]. Findings from extant literature indicate that PTSD affects 3.9% of the global population, with a higher prevalence among military personnel [2, 5, 6, 7]. The illness burden associated with PTSD is further exacerbated by the high level of medical and psychiatric comorbidities, including but not limited to treatment‐resistant depression [8, 9, 10].

Trauma‐focused therapy (TFT) [e.g., Prolonged Exposure (PE), Eye Movement Desensitization and Reprocessing (EMDR), and Cognitive Processing Therapy (CPT)] has established efficacy in reducing PTSD symptom severity and is recommended as first‐line treatment [2]. However, TFT has limited efficacy, accessibility, and is not always preferred by persons with lived experience [11]. In addition, other barriers to access (e.g., economic factors, stigma) further belie the population‐based impact of psychological treatments [11, 12, 13, 14, 15].

In addition to psychotherapeutic approaches, pharmacotherapy has been recommended for the treatment of symptoms and distress associated with PTSD [16]. Paroxetine and sertraline, both selective serotonin reuptake inhibitors (SSRIs), are FDA‐approved for the treatment of PTSD [17]. Other agents, such as other SSRIs (e.g., fluoxetine), antipsychotics and anticonvulsants, are often prescribed off‐label for persons living with PTSD [18, 19]. Notwithstanding, the overall average effect size in reducing psychopathology related to PTSD with the aforementioned on‐label and off‐label treatments is modest and adverse events, including but not limited to sexual dysfunction and weight gain, are treatment‐limiting [20, 21]. More recently, the US FDA rejected applications seeking authorization for the use of 3,4‐methylenedioxymethamphetamine (MDMA) and brexpiprazole in the treatment of adults with PTSD [22]. The aforementioned rejection, along with only two current FDA‐approved treatments for PTSD, underscores the urgent and unmet need to identify safe and effective novel therapeutics for persons with PTSD [23].

Ketamine is a noncompetitive N‐methyl‐D‐aspartate (NMDA) receptor antagonist [24, 25]. The modulation of glutamate, dopamine and norepinephrine by ketamine is posited to underlie ketamine's psychotropic effects, including mood elevation, attenuation of fear responses, alterations in sensory perception, and impairments in memory function. Preclinical and clinical evidence further indicates that ketamine enhances synaptic plasticity within the prefrontal cortex (PFC) and hippocampus (HPC), key regions involved in mood regulation and extinction learning [26]. Moreover, ketamine modulates various neurotransmitter pathways (e.g., opioidergic and neurotrophic) and exhibits analgesic, anti‐inflammatory, and antidepressant properties [27, 28]. Dysregulation of glutamatergic signaling has been implicated in the pathophysiology of PTSD, notably in processes such as memory consolidation and the encoding of traumatic experiences [17, 29, 30]. Taken together, the aforementioned pharmacologic profiles indicate that glutamatergic agents may be differentially effective in persons with major depressive disorder (MDD) and persons with trauma [31, 32].

Preliminary evidence suggests that ketamine may be efficacious in adults living with PTSD. In persons with Major Depressive Disorder (MDD) and a history of trauma, ketamine has demonstrated superior clinical improvement compared to traditional serotonergic antidepressants [32]. Moreover, in addition to alleviating overall distress, ketamine has also been shown to be successful in treating symptoms of irritability, agitation, dysphoria and anhedonia, all of which are highly prevalent in persons with PTSD [33, 34, 35, 36]. For example, ketamine is well established to be highly efficacious in addressing treatment‐resistant depression (TRD) and improving aspects of suicidality [25, 37]. Furthermore, symptoms (e.g., anhedonia, hopelessness and suicidal ideation) and underlying neural circuits implicated in the pathology of PTSD overlap with those observed in depression, providing the rationale for exploring whether ketamine is an efficacious and safe treatment in patients living with PTSD [38, 39, 40].

### 1.1 Aims of the Study

Herein, this review aims to critically evaluate randomized controlled trials (RCTs) documenting the effects of ketamine in persons with PTSD and synthesize potential neurobiological pathways that may subserve ketamine's effects in PTSD.

## 2 Methods

### 2.1 Search Strategy

This review followed guidelines outlined in the 2020 Preferred Reporting Items for Systematic reviews and Meta‐Analyses (PRISMA) [41]. A systematic search was conducted across OVID (Embase, MEDLINE, and PsycINFO) and PubMed databases from inception until July 15, 2025. The following Boolean search string was used to retrieve relevant studies: (“ketamine” OR “Ketalar” OR “Calypsol” OR “Kalipsol”) AND (“PTSD” OR “posttraumatic stress disorder” OR “posttraumatic stress symptoms” OR “trauma‐related stress”) AND (“randomized controlled trial” OR “controlled clinical trial” OR “randomized” OR “placebo” OR “drug therapy” OR “randomly” OR “trial” OR “groups”). Manual searches of the reference lists of the obtained articles were performed thereafter. Studies were limited to those administering ketamine intravenously and published in English.

### 2.2 Study Selection and Eligibility Criteria

The Covidence platform was utilized for the removal of duplicates and screening of relevant articles [42]. Two independent reviewers (L.Y. and A.L.) conducted title and abstract screening, followed by full‐text screening of articles. Only RCTs that administered intravenous ketamine to all participants were included. Furthermore, patients were required to be primarily diagnosed with PTSD without comorbidities other than depression. Ongoing clinical trials without available results were excluded. Any screening discrepancies were resolved via discussion between the reviewers.

### 2.3 Data Extraction

Extracted data were established a priori using a piloted data extraction table and included (1) author(s) and publication year, (2) participants, (3) intervention, (4) study assessments, and (5) outcomes of interest(s) (Table 1). Data extraction of relevant studies were conducted by two reviewers (L.Y. and A.L.). Given the heterogeneity of treatment regimes and outcomes, data were synthesized narratively, with studies grouped by key outcomes.

**TABLE 1:** Randomized controlled trials documenting the effects of ketamine on individuals with post‐traumatic stress disorder.

Author/year	Participants	Intervention: dosing regimen and duration	Study assessments	Outcomes of interest
Abdallah et al. (2022) [43]	158 participants with PTSD aged 18–70 were randomized to intravenous ketamine [low dose (*n* = 53); standard dose (*n* = 51)] or 0.9% saline (placebo) (*n* = 54).	Eight 40‐min infusions, twice weekly for 4 weeks. 4‐week follow‐up period. Low dose: 0.2 mg/kg Standard dose: 0.5 mg/kg	PCL‐5, CAPS‐5, CADSS, PANSS	*Clinical Outcomes*: – PCL‐5 scores improved for all groups over time (F_(9,133)_ = 37.1, *p* < 0.0001), but there was no treatment‐by‐time interaction (F_(18,137)_ = 1.1, *p* = 0.38). – CAPS‐5 scores also improved for all groups over time (*p* < 0.0001), and there was no treatment‐by‐time interaction (*p* = 0.07). – Neither low nor standard doses of ketamine significantly reduced PCL‐5 or CAPS‐5 scores compared to placebo (*p* = 0.04, adj. *p* = 0.11; *p* = 0.14, adj. *p* = 0.28, respectively) 24 h post first dose and at the end of the 4‐week treatment period. – There were no differences in PCL‐5 or CAPS‐5 scores between the ketamine treatment groups (*p* > 0.2, adj. *p* > 0.5). *Durability of Response*: – Participants' PCL‐5 scores showed a time effect (*p* < 0.001), no treatment main effect (*p* = 0.11), and no treatment‐by‐time interactive effect (*p* = 0.08) in the 4‐week post‐treatment period. – The low dose of ketamine produced significantly lower PCL‐5 scores compared to placebo (*p* = 0.01, adj. *p* = 0.03). *Adverse Events*: – CADSS found significant dose‐dependent dissociative symptoms as a result of ketamine treatment (*p* < 0.0001). – Dissociative symptoms reduced over the treatment period (*p* < 0.0001). – 80 min following the infusion completion, CADSS found that ketamine‐induced dissociative symptoms had dissipated, and PANSS showed that ketamine‐induced psychotomimetic symptoms had improved significantly. – Active groups reported more events of agitation, anxiety, irritability, and constipation.
Danböck et al. (2024) [44]	26 participants with PTSD. Participants were randomized to a ketamine (*n* = 12) or midazolam (*n* = 14) infusion. On average, participants receiving ketamine were aged 38.00 (10.35), and participants receiving midazolam were aged 36.21 (10.81).	A single infusion of either ketamine (0.5 mg/kg) or midazolam (0.045 mg/kg) over 40 min.	DSM‐5, fMRI scans	*Neurobiological Outcomes*: – Ketamine and midazolam groups did not differ in vmPFC‐amygdala RSFC, dmPFC‐amygdala RSFC, and amPFC‐amygdala RSFC during baseline. – Between groups, there were no significant differences in the change of vmPFC‐amygdala RSFC and dmPFC‐amygdala RSFC from baseline to the first, middle, and last 10 min of infusion. – From baseline to the first 10 min and last 10 min of infusion, the groups did not differ in the change of vmPFC‐amygdala connectivity. – The ketamine group showed a larger reduction in vmPFC‐amygdala RSFC from baseline to the middle 10 min of infusion compared to midazolam. – Within‐group changes from baseline to the same time point were not statistically significant in either group.
Duek et al. (2023) [45]	27 participants aged 24–63 with chronic PTSD were randomized to ketamine (*n* = 14) or midazolam (*n* = 13).	A single infusion of either ketamine (0.5 mg/kg) or midazolam (0.045 mg/kg). In the 4 days following infusion, all participants underwent exposure‐based psychotherapy and in vivo exposure (corresponding to the patients' avoidance behavior).	CAPS‐5, PCL‐5, MRI scans	*Clinical Outcomes*: – PTSD symptoms improved over time for both the ketamine and midazolam groups. – There was no significant difference between groups in the rate of improvement or the PCL‐5 score at the end of treatment. – Compared to symptoms at baseline, symptoms at the end of treatment and at 30‐day follow‐up improved significantly. – The improvement in PTSD symptoms was maintained at the 90‐day follow‐up. *Neurobiological Outcomes*: – The ketamine group had lower activation differences for traumatic versus relaxed memories compared to the midazolam group at the end of treatment. At the 30‐day follow‐up, this difference was only marginally significant. – The ketamine group had marginally lower hippocampal activation to trauma versus relaxed memory compared to the midazolam group at the end of treatment. At the 30‐day follow‐up, there was no difference between groups. – There were no differences between groups in vmPFC activation to traumatic imagery or sad imagery scripts compared to relaxed imagery at all time points (before treatment, after treatment, 30 days post‐treatment). – Between groups, functional connectivity between the amygdala and vmPFC was not significantly different at any time point. – In the ketamine group, functional connectivity between the amygdala and posterior hippocampus was significantly lower at the end of treatment compared to the midazolam group. There was no difference between groups at the 30‐day follow‐up. – The connectivity between the amygdala and the anterior hippocampus showed no group differences. – In the right UNC, ketamine showed a reduction in FA lasting at least 30 days compared to midazolam. Similar results were found in the left UNC, with a strong interaction between session and drug on FA.
Feder et al. (2014) [46]	41 participants aged 18–55 were randomized to receive either the ketamine (*n* = 22) or midazolam (*n* = 19) infusion first; participants then received the other infusion 2 weeks later. However, only 35 participants ultimately completed the study (6 after the first infusion and 29 after the second infusion).	Participants received a maximum of two infusions. In order to receive the second infusion, participants needed a CAPS score of ≥ 50 before the second infusion. Ketamine hydrochloride (0.5 mg/kg) and midazolam (0.045 mg/kg) infusions were administered over 40 min.	DSM‐IV, CAPS, IES‐R, CGI‐S and CGI‐I, CAPS, PRISE	*Clinical Outcomes*: – The ketamine group had lower IES‐R scores 24 h after the first infusion compared to the midazolam group; however, this result was only significant in seven patients who received ketamine first and one patient who received midazolam first. – Ketamine showed similar effects on all 3 PTSD symptom clusters (intrusion, avoidance, and hyperarousal). – CGI‐S and CGI‐I scores were significantly better following ketamine infusions. – Between treatment orders, the mean CAPS score 7 days after infusion did not differ significantly. *Durability of Response*: – Treatment and time had significant effects on IES‐R scores. *Adverse Events*: – Dissociative symptoms with ketamine peaked at 40 min after the start of infusion and resolved by 120 min. – No significant psychotic or manic symptoms were reported. – Frequent reported adverse effects of ketamine compared to midazolam according to PRISE in the first 24 h after infusion included: blurred vision (36% vs. 19%), dry mouth (21% vs. 16%), restlessness (23% vs. 10%), fatigue (21% vs. 23%), nausea/vomiting (21% vs. 3%), poor coordination (15% vs. 3%), and headache (13% vs. 13%).
Feder et al. (2021) [47]	30 participants aged 18–70 with chronic PTSD were randomized to ketamine (*n* = 15) or midazolam (*n* = 15).	6 infusions over 3 times a week for 2 weeks. 0.5 mg/kg of ketamine hydrochloride or 0.045 mg/kg of midazolam over 40 min.	DSM‐5, CAPS‐5, CGI‐S, CGI‐I, IES‐R, PRISE	*Clinical Outcomes*: – At baseline, total CAPS‐5 scores were similar in both the ketamine and midazolam groups. – At weeks 1 and 2, total CAPS‐5 scores were significantly lower in the ketamine group compared to the midazolam group. – In each of the four CAPS‐5 subscales, compared to midazolam, ketamine showed a significant treatment‐by‐time interaction for intrusion (*p* = 0.03), avoidance (*p* < 0.0001), and negative mood and cognitions (*p* = 0.02), but not for arousal and reactivity (*p* = 0.09). – The ketamine group had a significant treatment‐by‐time interaction on the CGI‐S (*p* = 0.009) and a significant main effect of treatment on CGI‐I scores (*p* = 0.004). – In a sample of 10 ketamine responders (≥ 30% symptom improvement 2 weeks after the first infusion), from baseline to 24 h after the first infusion, the mean change was −26.0 on the total IES‐R score. *Durability of Response*: – The median time for responders to lose response was 27.5 days after the primary outcome assessment day (2 weeks after the first infusion). – 2 participants did not lose response by their last assessments at 50 and 102 days, respectively, after their primary outcome assessments. *Adverse Effects*: – Transient dissociative symptoms emerged during ketamine infusions. – Significant psychotic and manic symptoms were not observed. – Using PRISE, after the start of infusions, the most commonly reported side effects of ketamine included blurred vision, dizziness, fatigue, headache, and nausea/vomiting. – During the assessment period, no suicidal behavior was recorded.
Norbury et al. (2021) [48]	21 participants aged 18–70 with chronic PTSD were randomized to ketamine (*n* = 11) or midazolam (*n* = 10).	Participants received 3 infusions of ketamine (0.5 mg/kg) or midazolam (0.045 mg/kg) each week for two weeks.	CAPS‐5, MRI scans	*Clinical Outcomes*: – Improvements in CAPS‐5 scores were observed in both groups, although the ketamine group showed greater improvement. *Neurobiological Outcomes*: – Increased connectivity between the vmPFC and amygdala during emotional face viewing was the strongest correlate of PTSD symptom improvement. – Patients with increases in rACC BOLD during negative emotional conflict regulation and greater task‐free (resting) connectivity between the rACC and anterior insula tended to have decreased PTSD symptom severity. – Stronger vmPFC‐amygdala connectivity during emotional face‐viewing predicted better treatment outcomes, especially in patients receiving ketamine. – In individuals who received ketamine, decreased dACC BOLD during emotional conflict and increased resting vmPFC‐anterior insula connectivity were associated with improvements in PTSD symptoms. – Data from both pre‐ and post‐infusion showed that post versus pre‐treatment shifts toward lesser face‐related excitation in the amygdala → vmPFC pathway and greater face‐related inhibition in the vmPFC → amygdala pathway led to greater improvements in PTSD symptom severity. – Only the ketamine group exhibited a relationship between improvements in PTSD symptoms and greater top‐down inhibition of the amygdala by the vmPFC. – vmPFC → amygdala inhibition had a stronger effect on PTSD symptom improvement in individuals who received ketamine compared to midazolam. – Lower baseline vmPFC‐amygdala connectivity during the emotional face‐viewing task was the strongest predictor of PTSD symptom improvement. – The relationship between baseline face‐related vmPFC–amygdala connectivity and later PTSD improvement was stronger in the ketamine group than in the midazolam group.
Pradhan et al. (2018) [49]	20 participants aged 24–60 with PTSD were randomized to receive a single infusion of ketamine combined with 12 TIMBER psychotherapy sessions (*n* = 10) or a single infusion of placebo (saline) combined with 12 TIMBER psychotherapy sessions (*n* = 10).	All participants received a single 0.5 mg/kg dose of either ketamine or placebo administered over 40 min. Mindfulness interventions were personalized according to participants' scores on ASMI. 3 mini‐TIMBER sessions were administered in the first week after infusion. Once participants relapsed, they underwent a full‐TIMBER session every week for 9 weeks.	CAPS, PCL	*Clinical Outcomes*: – There were no significant differences in baseline PCL between the two groups. – At 24 h, both groups experienced a significant remission of symptoms (> 60% reduction in PCL and CAPS scores; *p* < 0.0001). – Between groups, there was no significant difference in response. *Durability of Response*: – The ketamine group sustained a response for a significantly longer period compared to the placebo group (*p* = 0.022). – At relapse, PCL and CAPS scores were lower than pre‐treatment.

Abbreviations: amPFC, Anterior‐medial prefrontal cortex; ASMI, Assessment Scale for Mindfulness Interventions; BOLD, Blood‐oxygen‐level–dependent; CADSS, Clinician‐Administered Dissociative State Scale; CAPS, Clinician‐Administered PTSD Scale; CGI‐I, Clinical Global Impression–Improvement Scale; CGI‐S, Clinical Global Impression–Severity Scale; dACC, Dorsal anterior cingulate cortices; dmPFC, Dorsomedial prefrontal cortex; FA, Fractional anisotropy; fMRI, Functional magnetic resonance imaging; IES‐*R*, Impact of Event Scale–Revised; PANSS, Positive and Negative Syndrome Scale; PCL, Posttraumatic Stress Disorder Checklist; PRISE, The Patient‐Rated Inventory of Side Effects; PTSD, Post‐traumatic stress disorder; rACC, Rostral anterior cingulate cortices; RSFC, Resting‐state functional connectivity; TIMBER, Trauma Interventions using Mindfulness‐Based Extinction and Reconsolidation; UNC, Uncinate fasciulus; vmPFC, Ventromedial prefrontal cortex.

### 2.4 Quality Assessment

RCTs were assessed by two independent reviewers (L.Y. and A.L.) using the Cochrane Risk of Bias Tool for Randomized Studies (RoB2) (Table 2) [50]. All evaluative disagreements were resolved via discussion.

**TABLE 2:** Risk of bias/quality assessment of the included randomized controlled trials.

Study	Item						Quality rating
	1	2	3	4	5	6	
Abdallah et al. (2022) [43]	L	L	L	L	L	L	Good
Danböck et al. (2024) [44]	L	L	L	L	L	L	Good
Duek et al. (2023) [45]	L	L	L	L	L	L	Good
Feder et al. (2014) [46]	S	L	L	L	L	L	Good
Feder et al. (2021) [47]	L	L	L	L	L	L	Good
Norbury et al. (2021) [48]	S	L	L	L	L	L	Good
Pradhan et al. (2018) [49]	L	L	L	L	L	L	Good

Abbreviations: H, high risk of bias; L, low risk of bias; S, some concerns.

## 3 Results

### 3.1 Study Selection and Results

**FIGURE 1:**
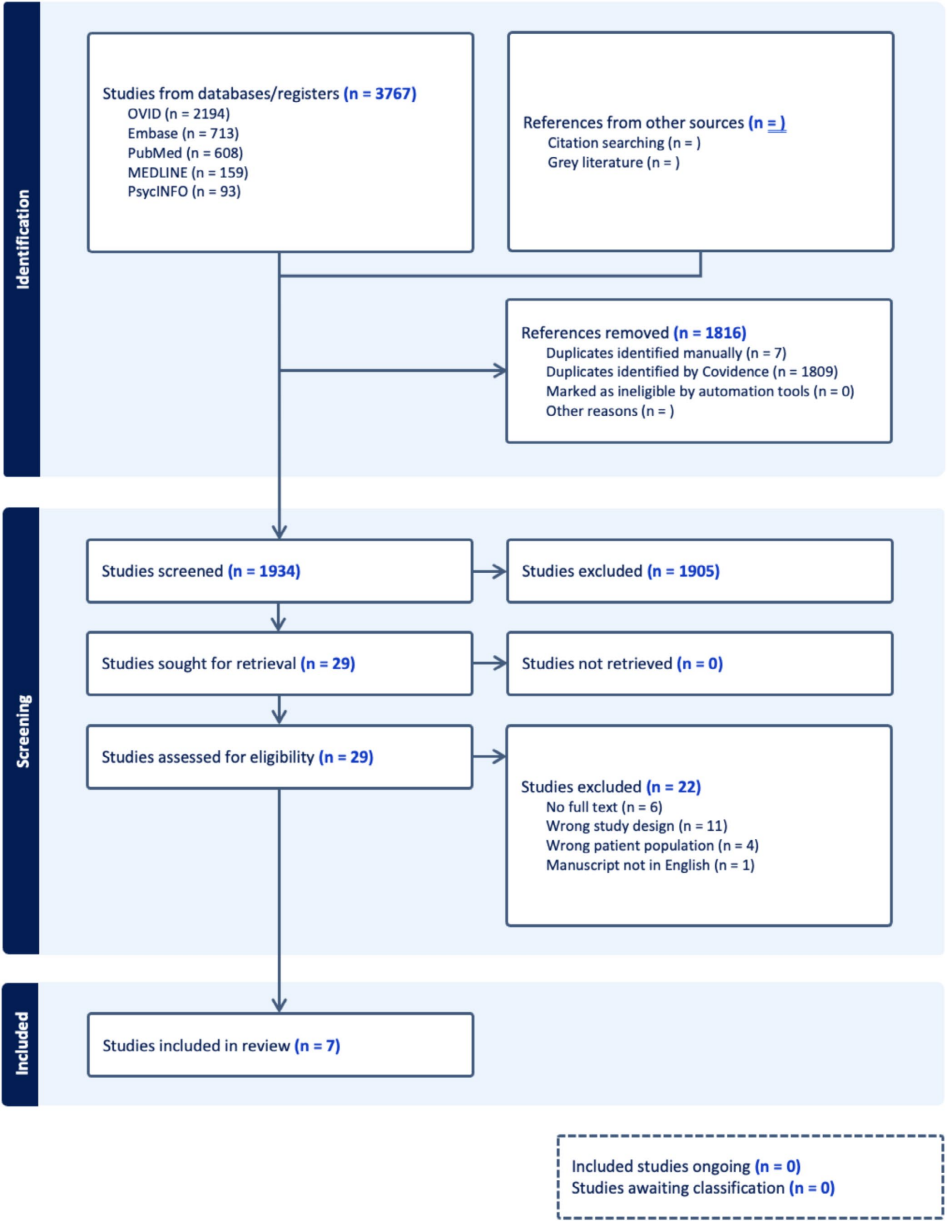
PRISMA flow diagram.

A total of 3767 studies were retrieved. 1809 studies were identified as duplicates and removed. Based on the eligibility criteria (Table 3), 1934 studies underwent title and abstract screening, and 29 studies were screened for full text. Studies were excluded for reasons including wrong study design (*n* = 11), no full text (*n* = 6), wrong patient population (*n* = 4), and manuscript not written in English (*n* = 1). Ultimately, seven studies were included in this systematic review (Figure 1).

**TABLE 3:** Eligibility criteria.

*Inclusion criteria*
Randomized controlled trials. Participants must be administered intravenous (IV) ketamine. All participants must be diagnosed with post‐traumatic stress disorder (PTSD). The primary outcome must evaluate ketamine's clinical efficacy in PTSD or investigate its underlying neurobiological mechanisms.
*Exclusion criteria*
Preclinical studies (i.e., animal studies). Participants must not have any comorbidities except for depression (e.g., anxiety). Non‐randomized studies (e.g., observational studies, case reports, reviews, meta‐analyses, post hoc analyses, editorials). Full‐text is unavailable or not in English.

### 3.2 Methodological Quality Assessment

All studies received a quality rating of “Good”. However, studies by Feder et al. [46] and Norbury et al. [48] contained methodological concerns regarding the generation of the random allocation sequence. Variations across studies (i.e., dosing regimens, outcome measures, follow‐up durations) also complicate interpretation, as some trials only assessed short‐term effects while others examined sustained response.

### 3.3 Overview of Included Study Characteristics

A total of 323 patients with PTSD were included across the seven included RCTs. Participants' ages ranged from 18 to 70, and the female percentage varied between 23% and 85.7%. All of the included RCTs investigated the administration of single or multiple subanesthetic doses of intravenous ketamine (0.2–0.5 mg/kg) to select participants; however, only select studies included a follow‐up period, which ranged from a few days to several months.

### 3.4 Clinical Outcomes of Ketamine in PTSD

We identified a total of six studies investigating the efficacy of ketamine in the treatment of adults with PTSD. In a trial administering eight low‐dose or standard‐dose ketamine infusions (0.2 or 0.5 mg/kg, respectively) over 4 weeks, both the treatment and placebo saline groups exhibited improvements on the Posttraumatic Stress Disorder Checklist (PCL‐5) (F_(9,133)_ = 37.1, *p* < 0.0001) and Clinician‐Administered PTSD Scale for DSM‐5 (CAPS‐5) (F_(1,124)_ = 103.4, *p* < 0.0001) from baseline to endpoint [43]. However, there were no significant treatment‐by‐time interaction effects observed for either measure [43]. Furthermore, at both 24 h after the first infusion and the end of the treatment period (4 weeks), there was no between‐group difference (ketamine vs. placebo) in PCL‐5 scores (*p* = 0.04, adj. *p* = 0.11; *p* = 0.14, adj. *p* = 0.28, respectively) [43]. Overall, rates of response (≥ 25% improvement in PCL‐5 scores) were not significantly higher in the treatment groups [43].

In a similar multi‐dose model where participants received six infusions of ketamine (0.5 mg/kg) or midazolam (0.045 mg/kg) over 2 weeks, a significant difference in total CAPS‐5 score was noted (F_(2,55)_ = 5.97, *p* = 0.0045) between treatment groups [47]. A significant reduction in CAPS‐5 score in favor of the ketamine group when compared to the midazolam control group at weeks one and two was noted [47]. In addition, there were more treatment responders (defined as ≥ 30% reduction from baseline to week two) in the ketamine group compared to the midazolam control group at week two [47]. The participants assigned to the ketamine group also exhibited greater clinical improvement, as indicated by a significant treatment‐by‐time interaction on the Clinical Global Impression–Severity Scale (CGI‐S) scores (*p* = 0.009) and a significant main effect of treatment on the Clinical Global Impression–Improvement Scale (CGI‐I) scores (*p* = 0.004) [47]. A comparable RCT, wherein participants were randomly assigned to receive six infusions of ketamine (0.5 mg/kg) or midazolam (0.045 mg/kg) over 40 min across 2 weeks, reported that participants assigned to ketamine exhibited a greater symptom improvement when compared to midazolam‐treated patients as evidenced by a change in the CAPS‐5 score [48].

A study investigating a single‐dose model with adjunct exposure‐based psychotherapy, compared ketamine (0.5 mg/kg) and midazolam (0.045 mg/kg), reported an improvemnt in PCL‐5 scores [baseline: 46.6 ± 13.35; 7 days post‐infusion: 33.8 ± 18.37; 30‐day follow‐up: 30.79 ± 15.79; 90‐day follow‐up: 31.04 ± 17.55] [45]. However, the symptoms of participants in the ketamine or midazolam groups both improved at the same rate, leading to considerable ameliorations from baseline to end of treatment and at the 30‐day follow‐up [45]. Another study that combined a single dose of ketamine with adjunct psychotherapy duplicated these findings, reporting that ketamine was unable to dramatically improve PTSD symptoms relative to placebo [49].

Lastly, in a crossover study where participants received a single infusion of ketamine (0.5 mg/kg) and a single infusion of midazolam (0.045 mg/kg), ketamine was observed to reduce symptoms across all three PTSD symptom clusters (e.g., intrusion, avoidance and hyperarousal) [46]. Furthermore, 24 h after the first infusion, the Impact of Event Scale‐Revised (IES‐R) scores improved more in those who received ketamine [46]. Crossover analyses also revealed that CGI‐S and CGI‐I scores were significantly greater following ketamine infusions [46]. However, between treatments, the mean CAPS and IES‐R scores 7 days after infusion did not differ significantly [46].

### 3.5 Durability of Ketamine Response in PTSD

A total of five studies examined the durability of the ketamine treatment effect on PTSD symptoms. Among groups receiving multiple doses of ketamine (0.2 and 0.5 mg/kg, respectively) or placebo, participants' PCL‐5 and CAPS‐5 scores in a four‐week post‐treatment period reported a time effect (*p* < 0.001; *p* < 0.0001, respectively), but no significant treatment main effect (*p* = 0.11; *p* = 0.26, respectively) nor treatment‐by‐time interactive effect (*p* = 0.08; *p* = 0.13, respectively) [43]. Notably, at the end of the post‐treatment period, substantially lower PCL‐5 scores were observed in the low‐dose group compared to placebo (*p* = 0.01, adj. *p* = 0.03) [43]. In contrast, PCL‐5 score differences between the standard‐dose group and placebo were not significant (*p* = 0.34, adj. *p* = 0.34) [43]. There were also no significant differences in CAPS‐5 score changes between all groups at the end of post‐treatment [43].

However, in a single‐dose model with adjunct exposure‐based psychotherapy, improvements in PTSD symptoms were maintained in both ketamine and midazolam groups at the end of the 90‐day follow‐up period [45]. In a separate study which combined pharmacotherapy and psychotherapy, the ketamine group sustained a response for a significantly longer period compared to the placebo group (*p* = 0.022) [49]. Additionally, a crossover design comparing ketamine and midazolam reported that treatment and time both had significant effects on IES‐R scores [46].

Finally, examining ketamine responders in a multi‐infusion study, the median time for responders to lose response after the primary outcome assessment day (2 weeks after the first infusion) was 27.5 days [47]. Notably, two participants did not relapse by their last assessments at 50 and 102 days, respectively, after their primary outcome assessments [47].

### 3.6 Adverse Effects of Ketamine in PTSD

We identified three studies that evaluated adverse effects associated with ketamine treatment in persons with PTSD as an additional outcome. Over multiple doses, participants with PTSD receiving low and standard doses (0.2 and 0.5 mg/kg, respectively) of ketamine exhibited an increased frequency of agitation, anxiety and irritability compared to placebo [43]. Furthermore, participants receiving ketamine exhibited Clinician‐Administered Dissociative States Scale (CADSS) measured dose‐dependent increases in dissociative symptoms (*p* < 0.0001) [43]. Notwithstanding, dissociative symptoms were often transient, decreasing in severity with subsequent treatments and were rarely a reason for discontinuation [43]. In addition, evidence indicated that broad‐based psychopathology did not worsen and, in fact, trended in the direction of improvement as measured by the Positive and Negative Syndrome Scale (PANSS) [43].

In a similar study where participants with PTSD received multiple doses of ketamine (0.5 mg/kg), transient dissociative symptoms occurred during infusions, but significant psychotic and manic symptoms were not observed [47]. Moreover, reported using the Patient‐Rated Inventory of Side Effects (PRISE), common side effects of ketamine included blurred vision, dizziness, fatigue, headache and nausea/vomiting [47]. No increase in suicidal behavior was reported during the assessment period [47].

Lastly, in a crossover model, dissociative symptoms with ketamine peaked at 40 min after the start of infusion and resolved by 120 min [46]. The most commonly reported adverse effects of ketamine compared to midazolam included blurred vision, restlessness, and fatigue. There were no significant psychotic or manic symptoms reported in the foregoing study [46].

### 3.7 The Effect of Ketamine on Mechanisms Implicated in the Pathophysiology of PTSD

We identified a total of three studies evaluating the neurobiological effects of ketamine on PTSD from the search. In a single‐infusion model, ketamine and midazolam groups did not differ in their change of ventromedial prefrontal cortex (vmPFC)‐amygdala, dorsomedial prefrontal cortex (dm‐PFC)‐amygdala, or anterior‐medial PFC (amPFC)‐amygdala resting‐state functional connectivity (RSFC) from baseline to the first and last 10 min of infusion [44]. However, from baseline to the middle 10 min of infusion, the ketamine group exhibited a larger reduction in vmPFC‐amygdala RSFC, but the within‐group changes from baseline to this timepoint were not statistically significant in either group [44]. Changes in dm‐PFC‐amygdala RSFC and amPFC‐amygdala RSFC exhibited no significant differences from baseline to the middle 10 min of infusion between groups [44].

In a similar study where participants received a single infusion of ketamine or midazolam, underwent psychotherapy, and listened to scripts detailing personal traumatic, sad, and relaxed events, vmPFC‐amygdala functional connectivity was not significantly different at baseline, during infusion, as well as 1 week and 30 days after infusion [45]. However, functional connectivity between the amygdala and posterior hippocampus (pHPC) was significantly lower at the end of treatment in the ketamine group [45]. This difference was also observed at the 30‐day follow‐up.

After treatment was completed, the ketamine group reported significantly lower amygdala activation and marginally lower hippocampal activation differences when recalling traumatic versus relaxed memories [45]. These effects were not maintained at the 30‐day follow‐up [45]. Ketamine also showed a reduction in fractional anisotropy (FA) in the right uncinate fasciculus (UNC), lasting at least 30 days compared to midazolam [45]. Similar results were found in the left UNC, with a strong interaction between session and drug on FA [45].

A multi‐infusion model comparing ketamine and midazolam groups revealed that increased connectivity between the vmPFC and amygdala during emotional face viewing had the strongest association with PTSD symptom improvement [48]. Patients with enhancements in rostral anterior cingulate cortices (rACC) blood oxygen level dependent (BOLD) during negative emotional conflict regulation, and greater task‐free (resting) connectivity between the rACC and anterior insula, tended to have reduced PTSD symptom severity [48]. In addition, stronger vmPFC–amygdala connectivity during emotional face viewing predicted better treatment outcomes, especially in patients receiving ketamine [48]. Moreover, in individuals who received ketamine, decreased dorsal anterior cingulate cortices (dACC) BOLD during emotional conflict and increased resting vmPFC–anterior insula connectivity were linked to improvements in PTSD symptoms [48].

In both groups, data from both pre‐ and post‐infusion showed that post versus pre‐treatment shifts toward lesser face‐related excitation in the amygdala → vmPFC pathway and greater face‐related inhibition in the vmPFC → amygdala pathway led to greater improvements in PTSD symptom severity [48]. Moreover, in all groups, symptom improvement was associated with lower face‐related amygdala → vmPFC excitation [48]. Remarkably, only the ketamine group exhibited a relationship between improvements in PTSD symptoms and greater top‐down inhibition of the amygdala by the vmPFC [48].

In a cotherapy model where participants were randomized to receive either ketamine or a placebo and undergo psychotherapy, there was no significant difference in mean basal D‐serine (DSR) plasma concentrations between groups [49]. Pre‐treatment higher basal DSR plasma levels correlated with increased PTSD severity (*p* = 0.07) and shorter treatment responses (*p* = 0.93) [49]. Furthermore, in participants receiving ketamine only, significant differences in response were observed between those with basal DSR plasma concentrations greater than or equal to 3.5 μM and those who had concentrations lower than 3.5 μM (*p* = 0.001) [49].

## 4 Discussion

Our results suggest that ketamine is efficacious in the treatment of PTSD, yet findings from this review remain inconclusive. Only one multi‐infusion study and a crossover study reported greater ketamine‐induced symptom improvements compared to midazolam; however, two studies indicate that a single infusion of ketamine paired with psychotherapy was not effective in improving PTSD symptoms over control groups. Five studies reported mixed findings on ketamine's durability, but receiving multiple lower infusions of ketamine (0.2 mg/kg) may be more effective in sustaining treatment effects than standard doses (0.5 mg/kg) or the combination of single‐dose ketamine combined with psychotherapy. Ketamine was also observed to induce dissociation, blurred vision, dry mouth, and restlessness symptoms, but not psychotic, manic, or suicidal ideation and behaviors. Taken together, these results highlight ketamine's potential to relieve PTSD symptoms rapidly, although ketamine's effectiveness may be dependent on treatment regimen and strategy.

This highlights inconsistencies between real‐world and RCT findings, suggesting that PTSD as a stand‐alone diagnostic entity may not be an identical biophenotype to PTSD comorbid with depression. In a multi‐infusion trial involving patients with PTSD and comorbid depression, standard doses of ketamine reduced depression symptoms significantly more than placebo [43]. These findings are consistent with results from a larger study of PTSD patients, many with comorbid depression, in which a greater number of intravenous ketamine infusions was significantly associated with decreased PTSD and depression symptoms [51]. However, results from our review indicated that ketamine does not consistently outperform placebo. A potential explanation for why ketamine monotherapy may be less efficacious in RCTs than in real‐world settings is the variation in off‐label use regimens (e.g., higher doses, increased frequency) in clinical practice. Thus, PTSD may require a distinct treatment protocol compared to TRD.

Currently, ketamine's mechanisms in treating PTSD are unclear [27, 52]; however, it has been hypothesized that ketamine modulates neurobiological processes that play a role in fear learning and fear extinction [53, 54]. Specifically, ketamine has been shown to modulate gamma‐aminobutyric acid (GABA), glutamate and glutamine neurotransmitter concentrations, engage molecular cascades that participate in neurogenesis, neuroplasticity, synaptogenesis, and dendritic spine thickening (e.g., Brain‐Derived Neurotrophic Factor and mammalian Target of Rapamycin (BDNF–mTOR) signaling) [55, 56, 57, 58, 59, 60, 61].

In addition, ketamine modulates hormones as well as immune responses, all of which contribute to fear learning and fear extinction [26]. A relationship was observed between top‐down inhibition of the amygdala by the vmPFC and symptom improvement after ketamine treatment, suggesting that ketamine may enhance vmPFC's control over the amygdala during emotional processing, reducing hyperarousal and fear responses [62]. The aforementioned finding is further supported by observations in participants assigned to ketamine, wherein baseline vmPFC–amygdala connectivity during a face‐viewing task was more strongly associated with later PTSD improvement than in the midazolam group. Additionally, ketamine reduced amygdala‐pHPC functional connectivity, potentially reducing threat‐memory binding and hindering the retrieval of contextual fear memories [63, 64, 65, 66].

Despite these promising findings, there are several limitations that affect the inferences and interpretations of our data. There is a limited quantity of RCTs evaluating ketamine's efficacy in the treatment of adults with PTSD, which may limit our ability to detect a clinically significant effect. Additionally, the methods that included studies implemented were heterogeneous in nature, consisting of varied treatment periods, ranging sample sizes, different total numbers of infusions, as well as distinct approaches to adjunct psychotherapy. Furthermore, all studies involving a single infusion of ketamine included cotherapy, which limits the ability to evaluate the effect of single‐infusion ketamine as monotherapy in persons with PTSD. Of the studies that included a follow‐up period, the duration ranged considerably, limiting the observation and evaluation of treatment‐emergent adverse events and ketamine durability. Long‐term effects of treatment were also not evaluated in the available studies. Finally, the preponderance of studies considered in this review used intravenous delivery of racemic ketamine. As a result, studies that administered the enantiomer S‐ketamine or other ketamine metabolites via alternative routes of delivery (e.g., intranasal) were not included.

## 5 Conclusion

Available evidence suggests that intravenous ketamine may be efficacious in the treatment of adults with PTSD. However, there is a need for replicated studies to assess the therapeutic effect of ketamine as monotherapy, as well as with adjunctive treatments, for PTSD. Moreover, long‐term studies are needed to evaluate the maintenance, durability and long‐term safety of ketamine for persons with PTSD. Future studies should endeavor to examine the pharmacodynamic effect of ketamine in addition to clinical outcomes to better investigate relevant mechanisms of action that may underlie its therapeutic effect in PTSD.

## Author Contributions

Liyang Yin and Roger S. McIntyre conceptualized the study and developed the methodology. Liyang Yin and Andy Lu conducted the investigation, curated the data, performed the formal analysis, and validated the findings. Liyang Yin also created the visualizations and drafted the original manuscript. Andy Lu, Gia Han Le, Christine E. Dri, Sabrina Wong, Kayla M. Teopiz, Heidi Xu, Roger Ho, Taeho Greg Rhee, Heidi Ka Ying Lo, Maria‐Christina Sioufi, Yang Jing Zheng, Hezekiah C.T. Au, Hernan F. Guillen‐Burgos, Bing Cao, and Roger S. McIntyre provided critical revisions. Roger S. McIntyre supervised the project execution. Liyang Yin and Roger S. McIntyre approved the final edits.

## Funding

The authors have nothing to report.

## Conflicts of Interest

Dr. Roger S. McIntyre has received research grant support from CIHR/GACD/National Natural Science Foundation of China (NSFC) and the Milken Institute; speaker/consultation fees from Lundbeck, Janssen, Johnson & Johnson, Alkermes, Neumora Therapeutics, Boehringer Ingelheim, Bristol Myers Squibb, Sage, Mitsubishi Tanabe, Purdue, Pfizer, Otsuka, Takeda, MindMed, Neurocrine, Neurawell, Supernus, Bausch Health, Axsome, Novo Nordisk, Kris, Sanofi, Eisai, Intra‐Cellular, NewBridge Pharmaceuticals, Viatris, Abbvie and Atai Life Sciences. Dr. Taeho Greg Rhee was supported in part by the National Institute on Aging (#R21AG070666; R21AG078972; R01AG088647), National Institute of Mental Health (#R01MH131528), National Institute on Drug Abuse (#R21DA057540), and Health Resources and Services Administration (#R42MC53154‐01‐00). Dr. Rhee serves as a review committee member for National Institutes of Health (NIH), Patient‐Centered Outcomes Research Institute (PCORI) and Substance Abuse and Mental Health Services Administration (SAMHSA) and has received honoraria payments from NIH, PCORI and SAMHSA. Dr. Rhee has also served as a stakeholder/consultant for PCORI and received consulting fees from PCORI. Dr. Rhee serves as an advisory committee member for the International Alliance of Mental Health Research Funders (IAMHRF).

## Data Availability

The data that support the findings of this study are openly available in the PubMed repository at https://pubmed.ncbi.nlm.nih.gov/ and/or the OVID repository at https://ovidsp.ovid.com/.
